# The Association of Self-Compassion and Social Networking Site Use With the Acceptance of Cosmetic Surgery Among Female University Students: A Cross-Sectional Study

**DOI:** 10.7759/cureus.97390

**Published:** 2025-11-20

**Authors:** Mohadeseh Eskandari, Nasim Khalili, Asieh Sadat Baniaghil, Fatemeh Mehravar

**Affiliations:** 1 Student Research Committee, Golestan University of Medical Sciences, Gorgan, IRN; 2 Counseling and Reproductive Health Research Center, Golestan University of Medical Sciences, Gorgan, IRN; 3 Epidemiology, Ischemic Disorders Research Center, Golestan University of Medical Sciences, Gorgan, IRN

**Keywords:** cosmetic surgery, female students, self-compassion, social networking, tendency

## Abstract

Introduction: The tendency and demand for cosmetic surgery have steadily increased over the past decade. Several factors may contribute to this trend, and understanding these factors is essential. The primary objective was to investigate the relationship between self-compassion, social network use, and the acceptance of cosmetic surgery among female students.

Methods: This cross-sectional study was conducted from July 3 to August 11, 2025, involving 363 female students at the Golestan University of Medical Sciences in Iran. The inclusion criteria comprised Iranian nationality and ownership of a smartphone. The study used a stratified sampling method (each college as a stratum) and a convenience sampling method. Data were collected using the Self-Compassion Scale and the Acceptance of Cosmetic Surgery Scale. Additionally, daily usage of social networking sites was assessed. Participants completed the online tools. Multiple regression analysis IBM Corp. Released 2017. IBM SPSS Statistics for Windows, Version 26.0. Armonk, NY: IBM Corp. tested associations between variables.

Results: The findings showed that the self-compassion mean score had an inverse and significant relationship with the mean score of the tendency towards cosmetic surgery (r = -0.195, p = 0.001). Furthermore, the mean score of usage showed a direct and significant relationship with the mean score of the tendency towards cosmetic surgery (r = 0.148, p = 0.005). Regression analysis further indicated that self-compassion (B = -0.552, p = 0.001) and age (B = -0.552, p = 0.001) were the only significant predictors of cosmetic surgery tendency.

Conclusion: This study demonstrated that female students with higher levels of self-compassion were less likely to desire cosmetic surgery. Conversely, female students who spent more time daily on social networking sites were more likely to consider cosmetic surgery. Educational policymakers and planners can utilize these findings.

## Introduction

Cosmetic surgery encompasses surgical procedures [1] and therapeutic techniques designed to maintain, reconstruct, or enhance a person's physical appearance [2]. It modifies typical body features to help individuals achieve a look they find more appealing [1].

Cosmetic surgery has become increasingly accessible [3]. According to the International Society of Aesthetic Plastic Surgery (ISAPS), there was a global increase of 3.4% in 2023, with 34.9 million aesthetic procedures performed, over 15.8 million surgical and 19.1 million nonsurgical. The demand for these procedures has steadily risen over the last decade, with a notable 40% increase in the past four years [4].

Research in Sanandaj, Iran, found that 19.1% of participants expressed a desire for cosmetic surgery [5], whereas acceptance among adults in Makkah and Medina, Saudi Arabia, was 54.2% [1]. As societal attitudes toward beauty and self-improvement evolve, the growing interest in cosmetic surgery highlights shifting perceptions regarding aesthetics and personal enhancement across cultures.

Cosmetic surgery is often viewed as a means to enhance beauty and boost self-esteem, potentially increasing life satisfaction [6]. However, it is vital to acknowledge the considerable physical and psychological risks involved [3]. Research has identified various complications linked to cosmetic procedures, including postoperative infections, thromboembolic events, skin necrosis, hemorrhage, syncope, pulmonary edema, fat embolism syndrome, bowel cavity perforation, intra-abdominal injury, seroma formation, and local anesthetic systemic toxicity [7]. Moreover, many women experience significant depression shortly after surgery, highlighting its profound emotional impact [8].

Understanding the motivations behind seeking cosmetic surgery and re-evaluating patients’ perceptions of results are essential [6]. One effective psychological approach for improving attitudes and emotional well-being is self-compassion, which encourages kindness and understanding toward oneself.

Self-compassion has emerged as a powerful approach for managing distressing thoughts and emotions, contributing significantly to mental and physical well-being [9]. Research indicates that fostering self-compassion can improve body image, particularly among individuals undergoing cosmetic procedures [10]. Adult patients post-rhinoplasty often experience lower levels of self-compassion [11], while women seeking cosmetic surgery report greater body dissatisfaction [12]. Conversely, self-compassion is a protective factor against body image concerns [13]. Interventions promoting self-compassion have been shown to enhance body appreciation and satisfaction while reducing self-criticism [14]. In recent years, researchers have increasingly focused on the rise of social networking [15].

At the same time, social media plays a central role in shaping modern perceptions of beauty. Social networking sites (SNSs) serve as public forums where individuals share images, videos, and text content [16]. While social media has both positive and negative aspects, it significantly affects body image perceptions [17]. Women who are interested in cosmetic surgery tend to engage more with appearance-focused social media [12], while self-compassion-focused or body-positive content can improve self-image [16].

Given the limited research on the relationships between SNS use, self-compassion, and cosmetic surgery [13], and the rising global prevalence of aesthetic procedures, the present study aimed to investigate the association between self-compassion, social network usage, and acceptance of cosmetic surgery among female university students.

These three variables were selected based on their strong theoretical and empirical relevance: self-compassion as a protective psychological factor that can reduce body-related shame and enhance body image, SNS use as a prominent sociocultural influence shaping beauty ideals, and cosmetic surgery acceptance as a behavioral outcome reflecting these psychological and social processes [14,18].

## Materials and methods

Study design and participants

This analytical cross-sectional study was conducted from July 3 to August 11, 2025, involving 363 female students at Golestan University of Medical Sciences (GOUMS) in Gorgan, northeastern Iran. This university is a state institution with students from across the country and consists of seven colleges.

The primary objective was to investigate the relationship between self-compassion, daily social network usage, and acceptance of cosmetic surgery among female students. All aspects of the study were reviewed in accordance with the STROBE (Strengthening the Reporting of Observational Studies in Epidemiology) statement.

The inclusion criteria included Iranian nationality and ownership of a smartphone. Students who did not have access to the internet were excluded.

Sample size

The sample size was estimated using G*Power software (version 3.1.9.4) based on the results of a study by Nerini et al. (2019) [18]. The calculation determined a minimum required sample size of 332 students. To account for a potential 10% attrition rate, the final sample size was increased to 365 students. A stratified sampling method was employed, with the seven faculties serving as the strata. Within each faculty, participants were recruited using a convenience sampling approach. 

Sampling

A stratified sampling method was employed to ensure that the sample accurately represented the target population. The seven faculties of the university served as strata, and participants were selected from each using a convenience sampling approach.

Data collection

Data were collected using a demographic-reproductive information form, the Self-Compassion Scale, and the Acceptance of Cosmetic Surgery Scale (measuring the tendency toward cosmetic surgery). Additionally, participants were asked about their daily social networking site (SNS) usage.

Demographic

This form consisted of items assessing participants’ age, marital status, residence, employment status, ethnicity, household income, education level, spouse’s education, parents’ education, and current semester.

Self-compassion scale (SCS-SF)

The self-compassion scale-short form (SCS-SF) is a 12-item instrument designed to assess self-compassion (Raes et al., 2011). The scale measures six components: self-kindness (2 items), self-judgment (2 items), common humanity (2 items), isolation (2 items), mindfulness (2 items), and over-identification (2 items). Each item is rated on a five-point scale ranging from “almost never” to “almost always.” Total scores range from 12 to 60, with higher scores indicating greater self-compassion. Research supports the reliability of the SCS-SF, with Cronbach’s alpha coefficients of 0.86 or higher. The SCS-SF demonstrated adequate internal consistency with Cronbach’s alpha ≥ 0.86 for the total scale in all samples. Due to having only two items per subscale, reliability for individual subscales is lower, with correlations ranging from 0.54 to 0.75. A near-perfect correlation with the long form SCS (r ≥ 0.97) confirms that the SCS-SF is a reliable alternative when assessing overall self-compassion [19].

Khanjani et al. (2016) examined the psychometric properties of the Iranian adaptation of the Self-Compassion Scale. The Cronbach’s alpha coefficients for the total scale and its subscales, self-kindness, self-judgment, common humanity, isolation, and mindfulness, were 0.79, 0.68, 0.71, and 0.86, respectively [20].

Daily social networking site (SNS) usage

To assess daily Social Networking Site (SNS) usage, participants were asked to report the estimated amount of time (in minutes) they spend on these platforms during a typical day. Furthermore, they were asked to identify the specific SNS on which they believe they spend the most time [13]. To gain a comprehensive view of their engagement, participants were also asked to indicate all the SNS they regularly access from a provided list, which included Telegram, Instagram, WhatsApp, YouTube, Pinterest, X (formerly Twitter), TikTok, Facebook, and an "Other" option for any additional platforms.

Acceptance of cosmetic surgery scale (ACSS)

The acceptance of cosmetic surgery scale (ACSS) was developed by Henderson-King and Henderson-King in 2005. It originally consisted of 26 items, later reduced to 15. The scale assesses attitudes toward cosmetic surgery through three subscales: intrapersonal (5 items), social (5 items), and consider (5 items). Each item is rated on a 7-point Likert scale ranging from 1 (strongly disagree) to 7 (strongly agree). The total score ranges from 15 to 105, with higher scores indicating greater acceptance of cosmetic surgery. The reliability of the original English version has been well-established. Cronbach’s alpha values reported for the subscales are 0.89 for the intrapersonal subscale, 0.91 for the social subscale, and 0.91 for the consideration subscale, with an overall alpha of 0.88 for the total scale. These values indicate excellent internal consistency and support the use of the ACSS as a reliable instrument for assessing attitudes toward cosmetic surgery in diverse populations [21].

Dehghan Manshadi et al. (2017) reported that the Cronbach’s alpha for the total ACSS was 0.94, and for the interpersonal, social, and consideration subscales, it was 0.86 and 0.92, respectively. The psychometric properties confirmed that the ACSS is a valid and reliable instrument for assessing cosmetic surgery tendencies in the Iranian population [22].

Data collection procedure

Data collection began after receiving ethical approval from the Ethics Committee of the Deputy of Research and Technology at Golestan University of Medical Sciences.

The study objectives were clearly explained to participants, who were assured of confidentiality and informed of their right to withdraw at any time. Written informed consent was obtained from all participants. The consent form, study instruments, and all related materials were administered via Porsline, an online survey software.

Participants completed the self-report questionnaires through an online platform. The survey link was distributed via an online platform. After confirming eligibility and providing consent, participants completed all study items and submitted their responses electronically.

Data were analyzed using IBM Corp. Released 2017. IBM SPSS Statistics for Windows, Version 26.0. Armonk, NY: IBM Corp. Descriptive statistics, including means and standard deviations for continuous variables and frequencies and percentages for categorical variables, were first calculated. To examine the relationships between self-compassion scores, daily social network usage, and acceptance of cosmetic surgery, the independent t-test was used to compare means between two groups, the one-way ANOVA was applied for comparisons across more than two groups, Pearson correlation was used to assess the strength and direction of associations between continuous variables, and linear regression analysis was conducted to identify predictors of self-compassion and acceptance of cosmetic surgery scores. All statistical tests were two-tailed, and a p-value < 0.05 was considered statistically significant. Analyses were performed in accordance with the STROBE guidelines for cross-sectional studies.

## Results

This study involved 363 female students with a mean age of 22.99 ± 4.28 years. The mean age of the husbands of the married participants was 32.10 ± 6.012 years. A significant majority of the students were single (75.8%), lived in urban areas (96.7%), and identified as Persian ethnicity. Approximately 40.5% of the participants reported having a history of cosmetic surgery. The most common educational level was a bachelor’s degree (51%). Additional demographic details are presented in Table 1. The participants’ semesters ranged from 1 to 16, with a mean of 4.55 ± 2.60.

**Table 1:** The demographic-reproductive characteristics among female students (n=363)

Variable	Category	N (%)
Marital status	Single	275 (75.8)
	Married	88 (24.2)
Place of residence	Urban	351 (96.7)
	Rural	12 (3.3)
Employment status	Employed	14 (3.9)
	Unemployed (student)	349 (96.1)
Ethnicity	Persian	212 (58.4)
	Turkmen	10 (2.8)
	Sistani	12 (3.3)
	Other	129 (35.5)
Household income	< 100 million IRR	264 (72.7)
	> 100 million IRR	81 (22.3)
	Missing*	18 (5)
Education level	Associate	21 (5.8)
	Bachelor	185 (51)
	Master	41 (11.3)
	Doctorate	31 (8.5)
	Professional doctorate	85 (23.4)
Spouse’s education	< Diploma	4 (1.1)
	Diploma	8 (2.2)
	Associate	5 (1.4)
	Bachelor	32 (8.80)
	Master’s degree or higher	39 (10.7)
Father’s education	< Diploma	62 (17.1)
	Diploma	105 (28.9)
	Associate	29 (8.0)
	Bachelor	80 (22)
	Master	85 (23.4)
	Missing	2 (0.6)
Mother’s education	< Diploma	78 (21.5)
	Diploma	110 (30.3)
	Associate	33 (9.1)
	Bachelor	87 (24)
	Master	52 (14.3)
	Missing	3 ( 0.8)
Cosmetic surgery history	Yes	147 (40.5)
	No	216 (59.5)
Faculty	Medicine	143 (39.4)
	Nursing and Midwifery	86 (23.7)
	Paramedical Sciences	80 (22)
	New Technologies	25 (6.9)
	Dentistry	28 (7.7)

The findings also showed that most students were members of Telegram (98.4%) and Instagram (77%). The majority of the time spent on social networks was on Telegram (51.2%) and Instagram (38.8%) (Table 2).

**Table 2:** The rational and absolute distribution of social networking site (SNS) use among female students (n= 363)

Variable	Categories	N (%)
SNS membership	Telegram	359 (98.4)
	Instagram	281 (77)
	WhatsApp	231 (63.3)
	YouTube	213 (58.4)
	Pinterest	121 (33.2)
	X (Twitter)	66 (18.1)
	TikTok	28 (7.7)
	Facebook	22 (6)
Most used SNS	Telegram	186 (51.2)
	Instagram	141 (38.8)
	Others/None	36 (10)
Daily use of SNS (hours)	< 0.5	23 (6.3)
	1–2	47 (12.9)
	2–3	108 (29.8)
	3–4	71 (19.6)
	4–5	59 (16.3)
	> 5	55 (15.1)

Across age groups, there were no significant differences in cosmetic surgery acceptance or total self-compassion scores. However, social media use was significantly lower among students aged 25 and older (p < .001). Among self-compassion subscales, self-judgment, isolation, and over-identification differed significantly by age, suggesting that younger participants experienced slightly higher levels of self-critical tendencies. The results indicate that the mean scores for total self-compassion, daily time spent on social networks, and total cosmetic surgery tendency were 38.41 ± 11.33, 3.33 ± 1.54, and 66.75 ± 20.93, respectively. The mean scores for self-compassion and the cosmetic surgery subscales are listed in Table 3.

**Table 3:** Comparison of self-compassion, social networking site (SNS) use, and acceptance of cosmetic surgery scores across age cohorts (n=363) * The one-way ANOVA

Variable	Subscale	Total Sample (n=363) Mean ± SD	Age Cohort <20 years (n=37) Mean ± SD	Age Cohort 20-24 years (n=234) Mean ± SD	Age Cohort ≥25 years (n=92) Mean ± SD	p-value*
Acceptance of Cosmetic Surgery	Intrapersonal	24.15±6.94	22.77±6.28	24.08±7.03	24.62±6.91	.404
	Social	21.30±8.20	20.44±7.67	21.00±8.34	21.87±7.87	.589
	Consider	21.30±5.79	20.77±6.13	21.01±6.35	21.34±5.57	.870
	Total score	66.75±20.93	64.00±17.20	66.13±19.99	67.80±18.88	.591
SNS use (Hours)		3.33±1.54	3.59±1.55	3.52±1.53	2.63±1.32	< .001
Self-compassion	Self-kindness	6.82±1.82	7.36±2.00	6.82±1.77	6.64±1.92	.143
	Self-judgment	6.03±1.90	6.55±2.06	5.86±1.86	6.35±1.91	.031
	Common humanity	6.72±1.74	6.75±1.88	6.76±1.67	6.60±1.87	.744
	Isolation	6.06±2.09	6.47±2.19	5.88±2.10	6.47±2.04	.038
	Mindfulness	6.98±1.79	6.97±2.06	7.01±1.73	6.91±1.89	.894
	Over-identify	5.80±1.99	6.20±2.08	5.59±1.81	6.27±2.31	.012
	Total score	38.41±11.33	39.91±8.88	38.49±7.84	39.27±8.55	.220

Self-compassion was significantly higher among married students (p = 0.046) and participants from high-income backgrounds (p = 0.001). SNS usage time was greater among single students (p = 0.009) and those from low-income families (p = 0.001). Students enrolled in associate degree programs reported higher SNS use compared to those pursuing a master’s degree (p = 0.011), and participants whose fathers held a diploma exhibited higher SNS usage (p = 0.036), while participants whose spouses had higher education levels showed lower SNS use (p = 0.049). Regarding cosmetic surgery tendency, urban students (p = 0.030) and participants of Sistani ethnicity (p = 0.007) showed higher scores. No significant differences were observed for other categorical variables (Table 4).

**Table 4:** The relationship between self-compassion, SNS use, and cosmetic surgery tendency according to socio-demographic categorical variables (n = 363) *the independent t-test ** The one-way ANOVA SNS: social networking site

Variables	Total self-compassion	SNS use time	Total cosmetic surgery tendency
	p-value		
Marital status*	.046	.009	.387
Income*	.001	.001	.425
Education level**	.037	.011	.530
Father’s education**	.527	.036	.754
Spouse’s education**	.077	.049	.745
Residence*	.256	.079	.030
Ethnicity**	.093	.523	.007

Students’ age demonstrated a negative correlation with SNS use (r = -0.218, p < .001) and a positive correlation with cosmetic surgery tendency (r = 0.162, p = .018). The spouse’s age was negatively correlated with SNS use (r = -0.512, p = .021) (Table 5).

**Table 5:** The correlation between self-compassion, SNS use, and cosmetic surgery tendency with continuous demographic variables (n = 363) *The Pearson correlation

Variables	Total self-compassion		SNS use time		Total cosmetic surgery tendency	
	r	p-value	r	p-value	r	p-value
Age*	.036	.600	–.218	< .001	.162	.018
Spouse’s age*	.288	.231	–.512	.021	.442	.051

Regarding the primary aim of the study, the total self-compassion mean score showed an inverse and significant relationship with the overall mean score of the tendency toward cosmetic surgery (r = -0.195, p = 0.001), as well as with all its subdomains: individual, social, and consideration. 

Furthermore, the mean SNS usage time had a direct and significant relationship with the overall mean score of the tendency toward cosmetic surgery (r = 0.148, p = 0.005), including all subdomains: individual, social, and consideration (Table 6).

**Table 6:** The correlations between self-compassion, its subscales, cosmetic surgery tendency, its subscales, and social networking site use time among female students (n=363) *The Pearson correlation

	Cosmetic surgery tendency								SNS use	
Variables	Individual		Social		Considerations		Total			
	r	p-value*	r	p-value*	r	p-value*	r	p-value*	r	p-value*
Self-kindness	–.148	.005	–.111	.036	–.134	.011	–.147	.006	–.065	.220
Self-judgment	–.090	.088	–.069	.194	–.058	.274	–.083	.118	–.171	.001
Common humanity	–.097	.065	–.093	.079	–.142	.007	–.118	.026	–.063	.229
Isolation	–.093	.076	–.138	.009	–.100	.059	–.123	.020	–.211	.001
Mindfulness	–.140	.008	–.149	.005	–.184	.001	–.172	.001	–.062	.241
Over-identification	–.088	.094	–.150	.005	–.106	.045	–.131	.014	–.216	.001
Total self-compassion	–.166	.002	–.179	.001	–.184	.001	–.195	.001	–.202	.001
SNS use	.098	.064	.169	.001	.130	.013	.148	.005	-------	------

The regression model indicated that approximately 11% of the variation in the tendency towards cosmetic surgery was explained by the study variables, including ethnicity, residency, age, self-compassion, and SNS use (F (5, 211) = 5.201, p < 0.001, R² = 0.112). 

Among the predictors, self-compassion (B = -0.552, p = 0.001, 95% CI [-0.881, -0.223]) was negatively associated with cosmetic surgery tendency, suggesting that higher levels of self-compassion are linked to lower interest in cosmetic surgery. Age (B = -0.552, p = 0.001, 95% CI [0.044, 0.832]) was positively related, indicating a slight increase in tendency with age. Other predictors, including SNS use, marital status, and educational level, were not statistically significant (Table 7).

**Table 7:** The regression model predicting cosmetic surgery tendency (n=363) *Analysis of variance (ANOVA) The regression test totaled self-compassion, age, educational level, social networking site use, and marital status. Dependent Variable: total cosmetic surgery

Model	Sum of squares	df	Mean square	R²	F	p-value*
Regression	8138.063	5	1627.613	.112	5.201	< .001
Residual	64466.215	206	312.943			
Total	72604.278	211				

## Discussion

This study primarily aimed to examine the relationship between self-compassion and daily social networking site (SNS) usage, as well as the acceptance of cosmetic surgery (tendency toward cosmetic surgery) among female students of Golestan University of Medical Sciences (GOUMS) in 2025. The results indicated that the mean score of self-compassion had an inverse and significant relationship with the mean score of cosmetic surgery acceptance. Additionally, the mean score of daily social media usage had a direct and significant relationship with cosmetic surgery acceptance.

In line with the findings of this study, previous research has shown that individuals who have undergone cosmetic surgery (rhinoplasty) tend to have lower self-compassion scores than those without such a history [11]. Another study demonstrated that self-compassion provides a unique perspective that challenges societal beauty standards and fosters a positive relationship with one’s body [10]. It highlights the journey from self-criticism to self-love, emphasizing the empowering effects of self-compassion in reshaping body image narratives and breaking free from social pressures. Self-compassion-based therapy has been shown to reduce negative body image [23]. Moreover, a brief (15-minute) self-compassion intervention reduced body shame, although it did not affect body image [24].

This growing body of evidence reinforces the significance of self-compassion in promoting a healthier self-perception and more positive body image. Exploring the interaction between self-compassion and the acceptance of cosmetic surgery requires attention to the multifaceted nature of beauty standards in modern society. For example, exposure to beauty-related TikTok content has been shown to negatively influence how young women perceive their appearance [25].

Consistent with the current study’s results, another investigation found that participants (aged 12 to over 50) who were influenced by social media platforms to pursue cosmetic procedures exhibited increased interest in such treatments [26]. Similarly, participants in another study reported that negative comments from others prompted them to consider cosmetic surgery [8]. The “Zoom Boom”, a term referring to the surge in aesthetic treatments during the COVID-19 pandemic, appears to reflect a genuine trend, as evidenced by the growing public interest in plastic surgery procedures [27]. Furthermore, the media plays a crucial role not only in promoting beauty standards but also in influencing individuals’ decisions to undergo cosmetic procedures [28].

Conversely, Australian women who have undergone cosmetic surgery reported that the “cosmetic surgery lifestyle” has become normalized in modern society [8]. Variations across studies may be attributed to factors such as age [28], the type of social media platform used [26], cultural norms [8], and social media content exposure related to beauty ideals [25,26].

The interaction between self-compassion, social media use, and cosmetic surgery acceptance underscores the complex interplay between psychological, social, and cultural dimensions of body image. As awareness increases regarding the psychological and physical effects of cosmetic procedures, it becomes increasingly important to promote self-compassion and critical awareness of societal beauty pressures.

Understanding the diverse influences of cultural context and social media can help foster healthier perceptions of beauty, ultimately supporting more informed and self-accepting decisions about cosmetic surgery among women. Therefore, research exploring the relationships among self-compassion, social media use, and acceptance of cosmetic surgery should continue to address the complex and evolving standards of beauty in contemporary society.

A recent three-group study comparing exposure to TikTok videos on beauty tips, self-compassion strategies, or travel destinations found that face-related appearance shame, anxiety, and negative mood were significantly higher in the beauty group than in the self-compassion group. Women exposed to beauty-related content reported more upward appearance comparisons and greater appearance-related thoughts. Moreover, self-compassion levels were significantly lower in the beauty group compared to the self-compassion group [25].

While the regression model was statistically significant, it is important to consider the magnitude of the observed relationships to avoid overinterpretation. The model explained approximately 11% (R² = 0.112) of the variance in the acceptance of cosmetic surgery. This indicates that while self-compassion and social networking site use are significantly associated factors, a substantial proportion of the variance is accounted for by other variables not included in our model. Furthermore, the correlations, though significant, were modest in strength. This underscores the complex, multifaceted nature of the decision to pursue cosmetic surgery, which is likely influenced by a wider array of psychological, social, and cultural factors beyond the scope of this study.

Our findings must also be interpreted within the unique socio-cultural context of Iran. Iranian society presents a complex interplay of traditional values and modern, globalized influences, particularly regarding beauty standards. While our study found a notable history of cosmetic surgery (40.5%), the motivations may differ from those in other populations. For instance, compared to the high acceptance rate (54.2%) reported in Saudi Arabia [1], where procedures might be more openly discussed, the pressures in Iran may be more internalized or driven by a specific focus on feature-enhancing surgeries like rhinoplasty, which is highly prevalent. The stringent local beauty ideals, often emphasizing specific nasal features, combined with the constant exposure to globalized beauty standards on social media, can create a unique pressure cooker for young Iranian women. This cultural lens suggests that the pathway from social media use and self-criticism to cosmetic surgery acceptance is not universal but is significantly shaped by local norms and social pressures, making the promotion of self-compassion a culturally relevant intervention.

Limitations

The cross-sectional design of the study limits the ability to establish causality. Additionally, the sample was limited to female students, which reduces the generalizability of the findings to the broader population and both sexes. Future research could examine. Furthermore, our measure of social networking site use focused on duration and platform type; future studies would benefit from differentiating between types of content consumed (e.g., appearance-focused vs. body-positive) and modes of engagement (e.g., active vs. passive use) to provide a more refined understanding of its relationship with cosmetic surgery acceptance. Additionally, the study did not include other validated psychological measures, such as scales for peer influence or body dysmorphic disorder, which may also significantly impact the acceptance of cosmetic surgery. Incorporating such tools in future research would provide a more comprehensive psychological profile. Data were collected through self-report questionnaires, which are subject to social desirability and recall biases, particularly for self-compassion and daily SNS usage. To mitigate this in future studies, researchers could employ triangulation methods, such as using smartphone usage tracker apps to obtain objective data on SNS engagement, alongside self-report measures.

## Conclusions

This study found that female students with higher levels of self-compassion are less likely to pursue cosmetic surgery. Conversely, those who spend more time on social networks tend to show a greater interest in cosmetic procedures. It appears that fostering self-compassion can serve as a protective factor against social media pressures that encourage people to consider cosmetic surgery.

Educational policymakers and planners can utilize these findings when developing initiatives that promote self-compassion among female students through workshops, seminars, and curriculum enhancements focused on emotional well-being. They can also incorporate these findings into programs that teach social media literacy, equipping students with the skills to critically evaluate online content and its impact on body image. Furthermore, establishing supportive networks within educational institutions can encourage students to engage in open discussions about pressures related to cosmetic surgery. These strategies can enhance health support and foster a positive self-image among students.
